# Exploring reward-related attention selectivity deficits in Parkinson’s disease

**DOI:** 10.1038/s41598-021-97526-7

**Published:** 2021-09-21

**Authors:** Matthew J. D. Pilgrim, Zhen-Yi Andy Ou, Madeleine Sharp

**Affiliations:** Department of Neurology and Neurosurgery, Montreal Neurological Institute, McGill University, Montréal, QC H3A 2B4 Canada

**Keywords:** Attention, Reward, Parkinson's disease

## Abstract

An important aspect of managing a limited cognitive resource like attention is to use the reward value of stimuli to prioritize the allocation of attention to higher-value over lower-value stimuli. Recent evidence suggests this depends on dopaminergic signaling of reward. In Parkinson’s disease, both reward sensitivity and attention are impaired, but whether these deficits are directly related to one another is unknown. We tested whether Parkinson’s patients use reward information when automatically allocating their attention and whether this is modulated by dopamine replacement. We compared patients, tested both ON and OFF dopamine replacement medication, to older controls using a standard attention capture task. First, participants learned the different reward values of stimuli. Then, these reward-associated stimuli were used as distractors in a visual search task. We found that patients were generally distracted by the presence of the distractors but that the degree of distraction caused by the high-value and low-value distractors was similar. Furthermore, we found no evidence to support the possibility that dopamine replacement modulates the effect of reward on automatic attention allocation. Our results suggest a possible inability in Parkinson’s patients to use the reward value of stimuli when automatically allocating their attention, and raise the possibility that reward-driven allocation of resources may affect the adaptive modulation of other cognitive processes.

## Introduction

Where we focus our attention plays a critical role in downstream cognitive processes^1^. The ability to automatically attend to some environmental stimuli over others allows us to adaptively filter incoming information. This necessarily influences what enters memory, what we learn about, and what we are subsequently drawn to in our decisions^1^. Given our limited cognitive resources, the ability to selectively attend to information that is behaviourally relevant is a key step towards aligning cognitive resource allocation with our goals^2,3^. Attention deficits have been repeatedly demonstrated in early Parkinson’s disease but whether these deficits reflect an inability to automatically prioritize the allocation of attentional resources to what matters most is unknown^4,5^. Meanwhile, early Parkinson’s disease, a stage when cognitive function remains relatively intact, is a time when subtle failures of selective attention allocation could potentially have an important impact on behaviour more broadly, by influencing downstream cognitive processes such as learning and memory.

Evidence for the attention deficits in Parkinson’s patients comes primarily from studies measuring overall attentional capacity^4,6,7^. Little work has examined whether the content or the focus of attention is altered by Parkinson’s disease. One important exception is the body of work on attention set-shifting in Parkinson’s patients^8–11^, which has examined the use of top-down control mechanisms to voluntarily guide attention allocation towards rewarding stimuli^12,13^. These studies have demonstrated that Parkinson’s patients are impaired at using top-down control strategies to guide their attention towards reward, and that they are especially impaired when they are required to shift the focus of their attention because of changing reward contingencies. However, these studies do not address the more rapid and automatic process by which some elements of the environment are selected for attentional processing over others.

Meanwhile, recent evidence has highlighted an important role for reward in guiding the automatic selectivity of attention^14–22^, a process that has been suggested to depend on striatal dopamine^23,24^. This bears particular relevance to Parkinson’s disease where dopamine-dependent reward processing is disrupted^25–30^. One approach to demonstrating the way in which reward can modulate the focus of attention has been to associate reward with stimuli that are not relevant to the task at hand, and instead function as distractors. Across several studies, it has been shown that the magnitude of reward associated with a distractor can enhance the degree to which it causes distraction, such that distractors associated with high reward are more distracting than those associated with low reward^14,15,19,22^. These findings support the notion that reward information in the environment can orient attention towards stimuli in a manner that is distinct from top-down or bottom-up control^18,31,32^, though not all studies using this type of paradigm have been able to rule out the possibility that mere stimulus selection history is driving the effect^23,24,33–35^. Neuroimaging studies looking to uncover the neural mechanisms of this process have suggested that the striatum, and more specifically, striatal dopamine, are involved^23,24,34,36^, and that reward-related activity in the dopaminergic midbrain is associated with enhanced representation of stimuli in sensory cortices^37^.

It is well established, across a number of different paradigms, that Parkinson’s patients have reduced reward sensitivity related to dopamine loss. Much of this work has focused on how this manifests as reduced learning from reward^25,26,28–30,38^. However, even reward sensitivity measured in isolation of any dependent cognitive process (e.g. the effect of reward on pupillary dilatation) is reduced in Parkinson’s patients^39,40^. This raises the possibility that altered reward sensitivity could have consequences on cognitive processing *beyond* its effect on learning and motivation. Indeed, there is evidence that reward helps guide the automatic prioritization of cognitive resource allocation across a number of domains, including working memory^41–43^ and episodic memory^44,45^, and that this reward-guided prioritization process is lost in Parkinson’s patients^46^. Whether the reward-guided allocation of attention resources is similarly impaired in Parkinson’s disease in a dopamine-dependent manner is not known.

To address this question, we used a standard two-stage attention capture task, which has been previously used to show the role of reward in guiding attention^14,15,22^. The first phase is a reward-association paradigm where different stimuli are paired with either a low or a high reward. The second phase is an attention test where the stimuli previously associated with reward now act as goal-irrelevant distractors to draw attention away from targets. Critically these distractors are also designed to be less salient than the targets. To assess the role of dopamine, we tested patients with Parkinson’s disease in a within-subject design, both ON and OFF dopaminergic medication, and compared them to older controls. As expected, we found that Parkinson’s patients exhibited a similar degree of distraction from the high-reward and the low-reward distractors. Contrary to our predictions, however, we did not find any evidence to support the possibility that dopaminergic replacement restores the effect of reward-associated distractors on attention, nor that dopaminergic replacement modulates the overall effect of distractors on attention: the degree of distractibility exhibited by patients both ON and OFF was similar across conditions. These findings suggest a possible inability in Parkinson’s patients to use reward information to selectively guide their attention, but leave open questions about whether this inability can be solely attributed to attentional mechanisms or whether learning deficits may also play a role.

## Methods

### Patients and control subjects

Forty-three Parkinson’s disease patients (13 females, mean ± SD age = 63.8 ± 6.4) and 31 control subjects (21 females, mean ± SD age = 63.8 ± 7.9) were recruited to participate in our study. Patients were recruited from the Movement Disorders Clinic at the Montreal Neurological Institute, community groups and the Parkinson Quebec Network, a registry of Parkinson’s patients interested in research who have been referred by movement disorder specialists. Control subjects were recruited from spouses and friends of patients, community groups and social media posts. None had major health issues, neurological disorders or active psychiatric problems. All subjects denied color blindness and confirmed their ability to distinguish colors. Disease duration ranged from 0.42 to 14.25 years (Mean years = 4.75 ± 3.25). All patients were taking levodopa, 6 patients were additionally taking a dopamine agonist (either pramipexole or rotigotine). See Table 1 for detailed demographic and clinical information. Comparing demographics across groups with Welch-approximated two-sample T-tests^47^ and Chi-squared tests, we note that patients had fewer years of education than controls (*p* = 0.031) and that there were fewer women in the Parkinson’s group than in the control group (*p* = 0.003). To control for these differences, we included sex and education as covariates in our analysis. All subjects gave informed written consent and were compensated for their participation. The study was approved by the McGill University Health Centre Research Ethics Board and all procedures were performed in accordance with the appropriate institutional guidelines.

**Table 1:** Demographic and neuropsychological information.

Measure	Parkinson's patients (N = 43)	Controls (N = 31)	*p* value
Age	63.8 (6.4)	63.8 (7.9)	.996
Education, years	15.2 (3.5)	17.1 (2.7)	.009**
Disease duration, years	4.8 (3.3)	NA	NA
Total PD medication, mg	622.3 (363.3)	NA	NA
Percent female	30%	68%	.003**
MoCA	26.7 (2.6)	27.8 (1.5)	.031*
Verbal fluency (MoCA)	12.4 (4.2)	13.8 (3.4)	.100
Digit span test	11.2 (2.3)	12.1 (2.0)	.077
Symbol digit modalities test	40.1 (10.7)	47.4 (8.5)	.002**
Geriatric Depression Scale	8.6 (6.1)	5.4 (5.4)	.114
Apathy Evaluation Scale	58.4 (8.2)	58.9 (12.2)	.825

MoCA = Montreal Cognitive Assessment, verbal fluency is taken from the Language section of the MoCA. Values presented are mean (SD).

*p < 0.05, **p < 0.01.

### General procedure and medication manipulation

All subjects came to the lab for two sessions and, to minimize practice effects, the interval between sessions was at least one and a half months. At both sessions, subjects completed the full neuropsychological battery (described below) and a behavioral task which was divided into two phases: the reward association phase and the attention test phase (described in detail below). All sessions were conducted in the morning, starting between 9 and 10 a.m. to allow us to control the timing of medications more easily and to control for circadian factors. For Parkinson’s disease patients, the OFF medication session was conducted after an overnight withdrawal (minimum 15 h) from dopamine medications, and the ON session was conducted with patients having taken their medication one hour prior to the start of testing. The order of these sessions was counterbalanced across subjects. Fifteen Parkinson’s patients did not complete their second session: eight missed their OFF session and seven missed their ON session. Three older controls did not complete their second session. All of these subjects were still included in the analysis such that we have 28 patients and 28 controls with both sessions, and 15 patients and 3 controls with only one session. See Supplementary Table 1 for demographic comparisons between the ON and OFF samples.

### Neuropsychological battery

All subjects were administered a neuropsychological battery to establish baseline cognitive functioning (Table 1). This battery included the Montreal Cognitive Assessment (MoCA)^48^, the Digit Span^49^ and the Symbols Digit Modalities Test (SDMT)^50^. Subjects were also administered the Geriatric Depression Scale^51^ and the Apathy Evaluation Scale^52^. Patients and controls were compared on their various neuropsychological scores using Welch- approximated two-sample T-tests^47^. Patients scored 1.1 points lower on the MoCA (*p* = 0.031) and 7.3 points lower on the SDMT (*p* = 0.002). The SDMT has been used in studies of other neurodegenerative patient populations as an overall marker of cognitive ability that is sensitive to decline^53,54^. For this reason, to account for differences in cognitive ability we chose to include performance on the SDMT in our models.

### Task

**Figure 1:**
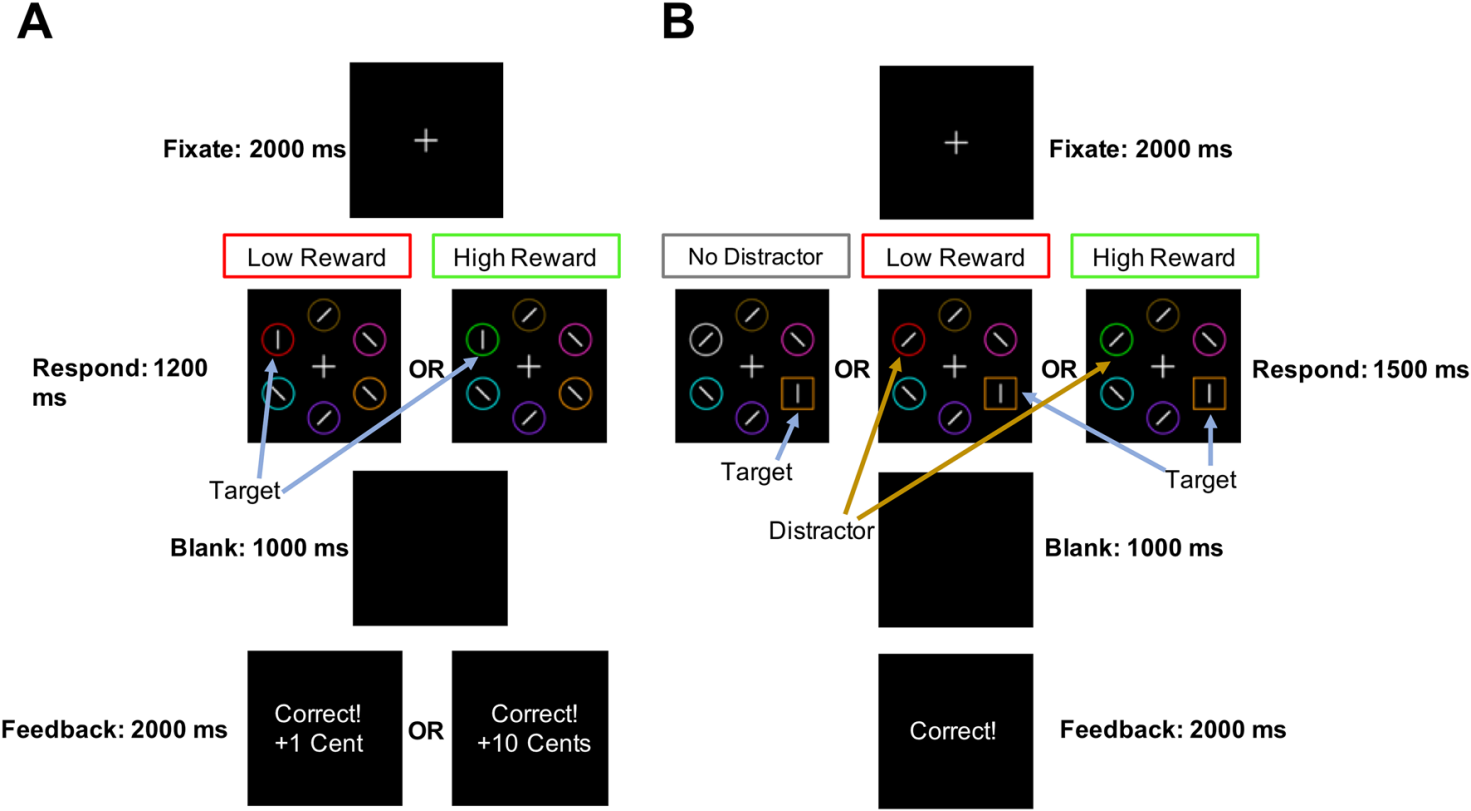
Trial sequence for the two phases of the task. (**A**) Reward association phase: Targets in the reward association phase were defined by a pre-specified color (e.g., red and green). Subjects reported the horizontal or vertical orientation of the white bar inside the target by pressing one of two keys. Correct answers were differentially rewarded based on the color (e.g., 10 cents for green and 1 cent for red). (**B**) Attention test phase: Here subjects were told to ignore the colors. Targets were identified as the unique shape. Once again, subjects reported the orientation of a white bar inside the target by pressing one of two keys. Half of the trials contained a distractor, i.e. a non-target shape in a color previously associated with a reward (e.g., green for the low reward distractor and red for the high reward distractor). The other half of the trials did not include a distractor.

We used a task that has been used to measure the influence of reward-associated distractors on attention^14,33^. We made two modifications to the task: we used an expanded set of stimulus colors so that subject could participate twice, and we increased the response windows to accommodate the older age of our sample. The task consisted of two phases: the reward association phase and the attention test phase (Fig. 1). Participants completed the same task at each session with the only difference being the color of the stimuli.

#### Reward association phase

During this phase, subjects gradually learned to associate different colors with different levels of reward. Each trial presented 6 circles (2.3° × 2.3° visual angle) of different colors surrounding a white fixation cross (0.5° × 0.5° visual angle). Two colors were assigned to be target colors (red and green for one session, or blue and yellow for the other session). On each trial only one circle was depicted in a target color, and subjects were asked to report the orientation of a white bar that was inside the target circle as either vertical or horizontal using a key press. They were told to press the “z” key if the bar was vertical and the “m” key if the bar was horizontal. The bars inside the targets were always vertical or horizontal while the bars in the non-target circles were tilted at 45° to the left or right. Every trial had *either* a red or a green circle. Target color assignment was counterbalanced across sessions. The set of colors included red, green, blue and yellow for the targets, and orange, pink, purple, cyan and brown for the non-target circles. The spatial location of colors was randomly assigned on each trial.

At each session, one target color was assigned the high reward and the other target color was assigned the low reward. The high reward color (e.g. green in Fig. 1A) yielded 10 cents for correct responses on 80% of the trials and only 1 cent for correct responses on 20% of the trials. The low reward color (e.g. red) led to a reward of only 1 cent on 80% of the trials and 10 cents on 20% of the trials. Feedback following incorrect responses consisted of “Incorrect!” printed in red text and “0 cents” earned. Subjects were told that they would receive a cash bonus equivalent to the winnings from 100 randomly selected trials. Subjects had 1200 ms to make a response and were asked to fixate on a white cross in the center of the screen for 2000 ms between each trial. If they did not respond before the time limit, they heard a loud beep and the text “Too slow!” was presented in white on the screen. Subjects performed 240 trials following 20 practice trials, and they were given the possibility of taking a break after 120 trials.

#### Attention test phase

To probe the influence of reward on attention, the previously rewarded colors (e.g. green and red in Fig. 1B) were used as distractors in the attention test. Subjects were explicitly told that colors no longer mattered and that they should instead focus on the shape of the stimuli. Colors used were red, green, blue, yellow, white, orange, pink, purple, cyan and brown. In this phase, subjects were required to report the orientation (horizontal or vertical) of a white bar in a target shape. They pressed the “z” key if the bar was vertical and “m” if the bar was horizontal. The target shape was identified as the unique shape: the square among circles or the circle among squares. The target shape type (circle or square) was randomly selected for each trial. On every trial, one target shape and five non-targets were arranged in a circle around the center of the screen. Non-target shapes also contained white bars but these were diagonally oriented at 45°. Subjects were notified if they were “Correct” or “Incorrect” with white text after making a response. Subjects had 1500 ms to make a key response and, if they failed to make a response, they saw “Too Slow!” in white text accompanied by an audible beep from the computer. They were asked to fixate on a white cross in the center of the screen for 2000 ms between each trial. Critically, there were three types of trials: trials with a high-reward distractor among the non-target shapes, trials with a low-reward distractor, and trials where no distractor was present. On high reward trials, the color of one of the non-target shapes corresponded to the high-reward color from the previous reward association phase the subject had just completed. On low reward trials, the color of one of the non-target shapes corresponded to the low reward color from the reward association phase. Finally, on trials where no distractor was present, none of the non-target colors corresponded to the rewarded colors. Following 10 practice trials there were 240 trials: 50% of trials included a distractor (25% were high reward, 25% were low reward), and the other 50% did not include a distractor. The order of these trials was randomized. Subjects were offered a break after 120 trials.

### Analysis

To compare performance across groups and conditions, statistics were computed using mixed effects linear and logistics regressions (R lme4 package^55^), performed in.

R version 3.6.3^56^. The general approach to model specification was as follows: we included random intercepts for subjects and random slopes for all within-subject variables and interactions^57,58^. Because the maximally specified models often failed to converge, we specified a variance components structure for the G matrix that assumes zero covariance between random effects and we removed random effects that prevented a given model from converging. An alpha cut-off of 0.05 was selected for all effects.

For the reward association phase, we compared accuracy and reaction times between reward conditions and groups. We performed either mixed effects logistic regressions with probability of a correct response on each trial as the dependent variable or linear mixed effects regressions with reaction time on each trial as the dependent variable. Reaction times were transformed using base-ten logarithms for the sake of normality. We ran three separate models for each of the two dependent variables: one in controls only, which included only reward level (low or high) as our main experimental variable; one in Parkinson’s patients which included reward level, medication state (OFF of ON) and their interaction (medication*reward); and one in all subjects that included reward level, disease (control or Parkinson) and their interaction (disease * reward). All models included session (first or second) to control for practice effects and Symbol Digit Modalities Test performance to control for general processing and motor speed, and because performance differed between groups. Models with both patients and controls additionally controlled for education and sex because of group differences but sex had to be removed from the logistic regressions due to convergence failures.

Analyses for the attention test phase followed an identical approach. We ran three separate models for each dependent variable: one in controls only, which included only distractor type (no distractor, low reward or high reward) as our main experimental variable; one in Parkinson’s patients which included distractor type, medication state (OFF or ON) and their interaction; and one in all subjects that included distractor type, disease (control or Parkinson) and their interaction. As above, all models included session (first or second), and Symbol Digit Modalities Test performance, and the model with both patients and controls also included education and sex as covariates to account for sample differences.

The categorical variable distractor type had three levels: high reward, low reward, and no distractor. Because we were principally interested in the differences *between* these levels, it was coded using two vectors, each with 3 levels: V1 (1 = low reward, 0 = high reward, − 1 = no distractor) and V2 (0 = low reward, 1 = high reward, − 1 = no distractor). As a result, the regression coefficient for V1 represented the difference in logRT between the low reward condition and the grand mean, and the regression coefficient for V2 represented the difference between the high reward condition and the grand mean. In order to test all three possible contrasts between the distractor levels (no distractor vs. low reward, no distractor vs. high reward, and low reward vs. high reward), we used the esticon function in R to compute weighted sums of the relevant coefficients as follows^59^: no distractor vs. low reward = 2 * βV1 + βV2; no distractor vs. high reward = βV1 + 2 * βV2; low reward vs. high reward = βV1 − βV2. We applied the same approach to test the contrasts between the condition * variable interactions.

We conducted follow-up analyses to evaluate overall distractibility where the three-level distractor type variable was collapsed into a new variable with only two levels (distractor present vs. absent). As above, we ran three separate models: one including only controls only, one including only patients, and one including both patients and controls.

### Hardware and software

All computerized tasks were conducted on a MacBook Air (13-inch, 2017) with a 13.3-inch screen (diagonal) a 1440 × 900-pixel resolution and a 60 Hz refresh rate. Responses were collected with the device’s built-in keyboard. Subjects sat approximately 50 cm from the display though they were instructed to take a comfortable position. Our behavioral task was coded in Python Version 2.7.

## Results

### Reward association phase

**Figure 2:**
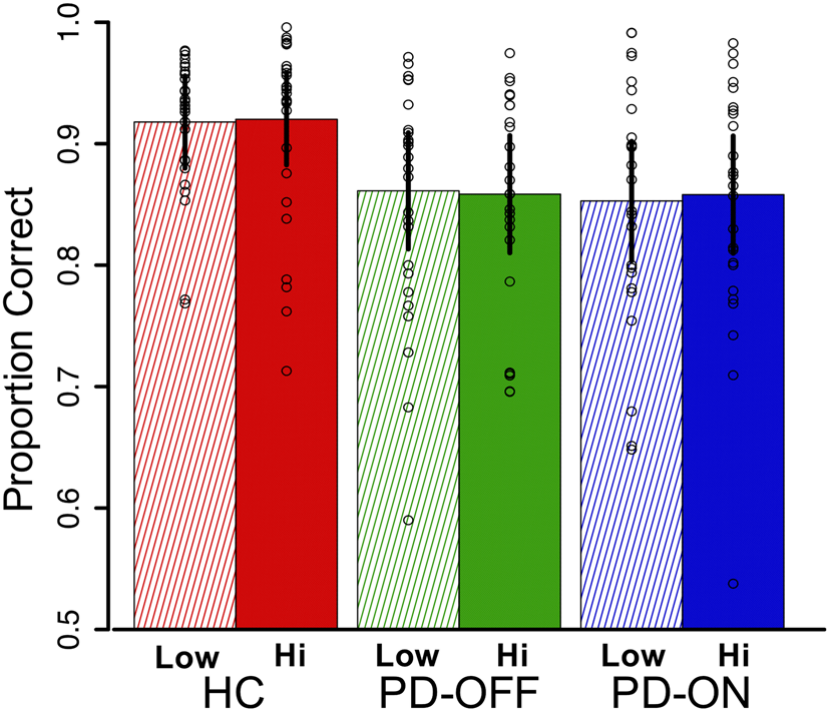
Performance during the initial color-reward association phase. Accuracy on the initial learning phase of the task, shown separately for trials where the target color was associated with a low reward upon correct responses versus trials where the target color was associated with high reward upon a correct response. Controls performed better than Parkinson’s patients (*p* < 0.001) but importantly, there was no effect of reward level on accuracy (HC: *p* = 0.907; PD: *p* = 0.553), nor was there a difference between groups in the effect of reward magnitude on performance (HC vs. PD: *p* = 0.136; ON vs OFF: *p* = 0.368). There was no effect of dopamine state on overall performance (*p* = 0.938). Error bars represent the standard error of the mean.

Results for the reward association phase are presented in Fig. 2 (accuracy) and model estimates, corrected and uncorrected, are presented in Supplementary Table 2 (accuracy) and Supplementary Table 3 (reaction time). As expected, Parkinson’s patients ON, OFF, and controls performed well. Reward magnitude of the feedback associated with the two target colors did not affect accuracy in either the controls (β_HC_ = 0.006, *p* = 0.907) or the patients (β_PD_ = 0.019, *p* = 0.553). Controls generally performed better than patients (β_HCvsPD_ = − 0.289, *p* < 0.001), but the influence of reward on accuracy was not different between the two groups (β_disease*reward_ = 0.029, *p* = 0.136). Dopamine medications did not influence accuracy (β_ONvsOFF_ = 0.005, *p* = 0.938) nor did they alter the influence of reward magnitude on performance (β_med*reward_ = 0.021, *p* = 0.368). For reaction time, reward magnitude had no influence in either controls (β_HC_ = − 0.004, *p* = 0.091) or patients (β_PD_ = 0.001, *p* = 0.623), nor did the influence differ between groups (β_PD_ = 0.001, *p* = 0.623). Dopamine medications did not influence reaction time (β_ONvsOFF_ = − 0.002, *p* = 0.591) nor did they interact with the influence of reward magnitude on reaction time (β_med*reward_ = 0.002, *p* = 0.420). The absence of a difference between groups for the effect of reward on both accuracy and reaction time is important for the interpretation of the second phase of the task as it indicates that patients (in both medication conditions) and controls had similar experiences during the reward training.

### Attention test phase

**Figure 3:**
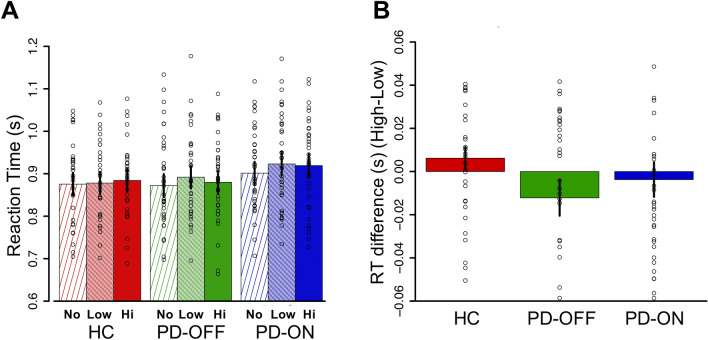
No influence of reward or dopamine on selective attention in Parkinson’s disease. (**A**) Reaction time (in seconds) on the attention task is shown for all three trial types (trials where no distractor was present, trials with a low-reward distractor and trials with a high-reward-associated distractor) for healthy controls and for patients ON and OFF medications. Parkinson’s patients were similarly slowed (i.e., distracted) by the presence of both low and high-reward distractors (low: *p* < 0.001, high: *p* = 0.006) and the degree of distraction caused by reward was not influenced by dopamine. (**B**) Difference in reaction time between the high-reward condition and the low-reward condition is shown for healthy controls and for patients ON and OFF medications. Error bars represent standard error of the mean (SEM).

Results from the attention test phase are presented in Fig. 3 (reaction time), mean reaction times are presented in Supplementary Table 4 and model estimates, corrected and uncorrected, are presented in Supplementary Table 5 (reaction time), 6 (reaction time) and 7 (accuracy). We were principally interested in measuring the effects of reward-associated distractors and of dopaminergic medications on attention in Parkinson’s patients. First, we found a main effect of distractors: Parkinson’s patients were slowed by the presence of both low and high reward distractors (low reward versus no distractor difference estimate = 0.010, *p* < 0.001; high reward versus no distractor difference estimate = 0.006, *p* = 0.006). However, there was no difference in slowing between low and high reward distractor trials (low vs. high difference estimate = 0.004, *p* = 0.170). Next, we examined whether the interaction between medication state and reward level of the distractor. Surprisingly, dopamine medications did not influence the effect of reward on attention in patients. Specifically, there was no difference between ON and OFF patients in the effect of low versus high reward distractors on reaction time (difference estimate = − 0.002, *p* = 0.546), low reward versus no distractor (difference estimate = 0.001, *p* = 0.757), or high reward versus no distractor (difference estimate = 0.002, *p* = 0.313). The only difference between patients ON and OFF was that patients ON were slower, across all three trial types, than patients OFF (β_ONvsOFF_ = 0.008, *p* = 0.006). This suggests that while there was an effect of medication on response time, it was not selective to distraction.

To better understand these results, we also examined the effect of reward on attention within each group separately. In healthy controls, we found that though the effect of reward level on attention did not reach statistical significance (difference estimates: low vs no distractor = 0.001, *p* = 0.530; high vs. none = 0.004, *p* = 0.100, low vs. high = − 0.002, *p* = 0.376), the overall direction of the effect was as expected: controls were slowest on the trials that included a high-reward distractor (881 ms ± 235), compared to the trials with a low-reward distractor (875 ms ± 233) and those without a distractor (871 ms ± 230). Furthermore, the degree of slowing induced by the highly-rewarded distractor was comparable to that reported previously in young controls^60^. In contrast, Parkinson’s patients OFF were significantly slowed only by the low-reward distractor (low vs. none difference estimate = 0.009, *p* = 0.005) and patients ON were slowed by both the low- and high-reward distractors, but to a similar degree (difference estimates: low vs. none = 0.01, *p* = 0.001; high vs. none = 0.008, *p* = 0.008; low vs. high = 0.002, *p* = 0.587).

In order to measure effects of disease on reaction time, we also compared patients to controls. Overall, patients were not slower than controls (β_HCvsPD_ = 0.001, *p* = 0.863). With respect to the effects of interest, Parkinson’s patients were more distracted than controls by the presence of a low reward distractor (low vs. no distractor difference estimate = 0.004, *p* = 0.008). However, this pattern was not observed in the presence of a high reward distractor (high vs. no distractor difference estimate = 0.001, *p* = 0.423). As noted above, all models controlled for session and performance on the Symbol Digit Modalities Test. Overall, across both groups, participants were faster on the second session (β_session_ = − 0.012, *p* < 0.001), and better performance on Symbol Digit Modality Test was associated with faster responses (β_SDMT_ = − 0.002, *p* < 0.001).

We were also interested in the effect of disease and dopaminergic medications on overall distractibility. To examine this, we collapsed across reward levels and compared reaction times on trials with a distractor to reaction times on trials without a distractor (Supplementary Table 8). We found that patients were more distractible than controls (β_disease*distraction_ = 0.001, *p* = 0.035), however, we did not find an effect of dopaminergic medication on distractibility (β_med*distraction_ = 0.001, *p* = 0.417).

Though we were primarily interested in reaction time, we also examined accuracy. There was no effect of reward level on accuracy in controls (β_V1_ = − 0.030, *p* = 0.542; β_V2_ = 0.004, *p* = 0.932) nor in patients (β_V1_ = − 0.005, *p* = 0.987; β_V2_ = − 0.053 *p* = 0.890). There was no effect of disease on overall accuracy (β_disease_ = − 0.073, *p* = 0.445), nor on the effect of rewarded distractors on accuracy (β_V1*disease_ = 0.015, *p* = 0.652; β_V2*disease_ = − 0.031 *p* = 0.350). There was no effect of dopamine medications on overall accuracy (β_med_ = − 0.048, *p* = 0.306), nor on the effect of rewarded distractors on accuracy (β_V1*med_ = 0.055, *p* = 0.145; β_V2*med_ = − 0.048 *p* = 0.202).

Finally, due to known links between mood symptoms and reward sensitivity deficits in Parkinson’s disease^40^, we conducted exploratory analyses examining the relationship between apathy and depression symptoms (measured with the Apathy Evaluation Scale and the Geriatric Depression Scale respectively) and the extent to which reward caused distraction in patients (Supplementary Fig. 1). We did not find any significant relationship between apathy and reward-driven attention (ρ_OFF_ = 0.15, *p* = 0.385; ρ_ON_ = − 0.28, *p* = 0.077), nor between depression and reward-driven attention (ρ_OFF_ = − 0.09, *p* = 0.598; ρ_ON_ = 0.12, *p* = 0.463), where reward-driven attention was taken as the reaction time difference between Hi and Low-reward distractors.

## Discussion

Reward is known to exert an automatic, involuntary effect on the allocation of attentional resources^14,15,22^. In Parkinson’s disease, both reward sensitivity deficits^25,26,40^ and attention impairments are well established^4,61^. However, little is known about whether the reward deficits in patients directly contribute to poor attention, and more specifically, whether Parkinson’s patients suffer from a dopamine-dependent inability to use reward to selectively allocate their attention resources. In a task where the presence of reward-associated distractors was used to probe the automatic reward-driven allocation of attention, we found that Parkinson’s patients were not influenced by levels of previously-associated reward information when allocating their attention; high reward-associated stimuli did not lead to greater attention capture than low-reward stimuli in patients. However, contrary to our predictions, we did not find evidence to support the possibility that this lack of a reward effect was due to dopamine deficiency. This absence of reward-driven attention allocation leaves open the possibility that an inability to use reward information may play a role in the attentional deficits seen in Parkinson’s disease.

These findings are broadly consistent with the body of work showing that patients are impaired at goal-directed attention allocation^9–11,62,63^. Goal-directed, or top-down control of attention has typically been studied in Parkinson’s patients by measuring attentional set formation^8–11^. In the typical task used, participants are explicitly aware of which stimuli they must attend to (the attentional set) and successful performance depends on the ability to adequately form, maintain, and switch attentional sets according to those explicit task goals. The current study focused instead on the more automatic, or involuntary, mechanism by which attention is allocated to behaviourally relevant stimuli. Such a mechanism is essential for matching the available cognitive resources to the nearly infinite amount of information constantly generated by our environment, not all of which is goal-relevant at a given time. In order to disentangle reward-guided attention from top-down goal-directed modulation, we used a task where reward was exclusively paired with distractors and was therefore in direct opposition to task goals. Previous studies using this task have found a relationship between striatal function and attention selectivity in healthy individuals^23,24,34^, suggesting that loss of normal striatal reward signaling in Parkinson’s patients may underlie the loss of reward-guided attention selectivity.

Though a relationship between striatal dysfunction and an inability to use reward to guide attention allocation seems highly plausible in Parkinson’s patients, the exact mechanism linking the two is less clear. One possibility is that, given known reinforcement learning deficits in Parkinson’s disease^25,26,28–30,38^, patients do not learn the reward values of the color-stimuli in the first place. This could then lead to a blunting of the differential levels of attention capture caused by the colors. While it seems likely that reward learning deficits could generally impact selective attention in Parkinson’s patients, we found no definitive evidence in the current task that this was the case. First, to ensure that learning deficits would not interfere, the learning phase was designed to be easy such that exposure to the different levels of rewarding feedback (offered only on correct trials) would be high, and similar across groups. Second, though a notable limitation of the task is that it did not allow us to specifically assess the strength of the color-reward associations formed in the first phase, groups did not differ in the effect of reward level on accuracy and reaction time, indicating that Parkinson’s patients had a similar experience to controls during the reward association phase. While it is possible that neither group learned the reward associations, previous work has shown that reward effects can be present during the test phase of the task without being present in the association phase^14^. Another possibility is that the impairment occurs at the stage of perceptual processing of the stimuli. Indeed, reward is thought to modulate attention by facilitating the perceptual processing of reward-associated stimuli, an effect that appears to depend on striatal activation *while* the selective allocation of attention is occurring, i.e. after the initial learning of the reward value has occurred, and at a time when the reward information is no longer relevant to the task^37^. Though exactly how this enhanced (or in the case of patients, possibly blunted) perceptual processing occurs is not clear, it has been proposed that the striatum plays a role in gating of information through the balance of activation in direct and indirect pathways^64–67^. This gating mechanism has mainly been suggested to explain the flow of information into and out of working memory, but could also have implications for the automatic selection of information to be processed in attention in Parkinson’s disease^68^. One possibility is that dopamine deficiency produces a deficit in reward-oriented gating of attention such that rewarding signals which normally “open” the gate no longer have such an effect. This could explain why patients do not allocate their attention more towards high reward than low reward stimuli. However, potentially complicating the above interpretation that the striatum is involved in reward-guided attention allocation is the fact that we did not find an effect of dopamine replacement on reward-guided attention in patients.

That we found no evidence to support the possibility that attentional selectivity is influenced by dopamine replacement was unexpected. We think this should be interpreted in the specific context of Parkinson’s disease, where the effects of dopamine on cognition are quite complex, and does not necessarily generalize to the role of dopamine in healthy brain function. One possible explanation is that top-down, goal-directed attention deficits, and in particular the ability to *shift* attention, which is known to be impaired in Parkinson’s patients^9,11^, might have made it more difficult to detect the more subtle effects of reward on attention allocation in this task. Attentional shifting deficits have been shown both in the OFF and ON medication state: patients OFF tend to perseverate on previously relevant stimuli^8^, whereas patients ON tend to have difficulty shifting their attention towards previously irrelevant features^10^. Inherent to our task was the requirement to explicitly shift attentional focus: from colors during the learning phase to shapes during the test phase. It is therefore possible that a tendency in the patients OFF to perseverate on the now irrelevant colors and an inability in the patients ON to shift their attention to the previously irrelevant shapes impacted performance during the test phase, and that the presence of these top-down attentional control deficits overrode the more subtle dopamine-dependent effects of reward on automatic attention allocation. Thus, it is possible that both reward-driven selectivity and top-down attentional set formation are impaired in Parkinson’s patients. Another possible explanation is that dysregulation of other neuromodulator systems contributes to selective attention deficits in Parkinson’s patients, and possibly even interacts with the deficits caused by dopaminergic loss, making it harder for dopamine replacement alone to remediate impairments. For instance, loss of cortical cholinergic projections from the basal forebrain is known to occur early in the disease and has been associated with attentional deficits^69,70^. In particular, the ability to resist distraction appears to be dependent on cholinergic integrity more so than on dopaminergic integrity^71^. Consistent with this, we found that dopamine state did not influence attention capacity, measured here as overall distractibility. It will be important for future studies to use tasks where resistance to distraction (i.e., top-down attentional control) is not in direct opposition to reward-driven attentional selectivity in order to obtain more sensitive measurements of automatic attention allocation, and to consider pharmacological manipulations of other neurotransmitter systems to attempt to triangulate the specific contributions of each.

It is important to note that in our sample of older controls, we did not find the effect of reward magnitude on attention selectivity that has been shown in younger controls using this task^14,15^. Though the difference in the slowing of responses between the high reward and low reward conditions was not statistically significant, it is worth noting that this difference (6 ms) was of similar magnitude to that reported previously in young controls^14,15,33,60^. However, as is typical of older adult samples, the variability was considerably higher, suggesting that we were underpowered to detect the desired effects. A weaker and more variable effect of reward could arise from two possible sources: first, an age-related reduction in sensitivity to rewarding outcomes has been demonstrated across a number of different cognitive processes including learning and memory^46,72–74^ and has been suggested to be caused by a decline in midbrain dopaminergic function^75^, and age-related differences in the power of monetary rewards to act as incentives^76–78^, which we used to drive reward-color associations as has been done previously. Given that the mean age of both our patient and control samples was over 60, we cannot discount the possibility of age-related blunting of reward sensitivity. To our knowledge, reward-driven attention has not previously been tested in aging populations so future work might shed light on this question.

There are several inherent limitations to the task we used that are important to note (see Le Pelley et al. (2016) and Sha and Jiang (2015) for a more in-depth discussion). First, as discussed above, the task design assumes that the reward values of the colors are successfully learned in the first phase. When, in a case like ours, distraction does not appear to be influenced by the magnitude of reward, it is difficult to pinpoint the exact mechanism for the reward insensitivity, which could stem from reward learning deficits (i.e. not learning the stimulus values in the first place) and/or from attentional processing deficits. Future work could consider using a more explicit reward association procedure in the first phase while still taking care to associate the reward with the stimuli rather than the actions, as this is a key feature of the task. Second, there is a confound between reward and attention history in the test phase of the task. The reward-association phase had subjects guiding their attention towards two specific colors repeatedly. It is therefore possible that the slowing of reaction times induced by the presence of those two colors (i.e. the reward-associated distractors) in the attention test phase is merely due to the distractors retaining their “target” status. To get around this, we specifically compared the slowing induced by the high and low reward-associated distractors, since they have the same “target” history. Previous studies using this task have been mixed in their ability to rule out the possibility that attention history is the main driver of the effect^31^, which suggests that the reward-capture effects induced by the initial reward-association phase are small. One possible modification to the task to consider in future work would be to include a third target color in the reward-association phase that is not paired with reward but nonetheless acquires the same prior target history. This unrewarded color could then serve as a second control condition during the test phase of the task. Related to the above points is also the fact that the task we used, and others like it, tend to elicit relatively small effect sizes^14,15,33,41,43,60^, especially when compared to those related to top-down control of attention, detailed above. In our case, as in previous reports of this task, changes in reaction time were on the order of ten or twenty milliseconds. Effect sizes of this magnitude are difficult to detect in populations whose cognitive behavior is inherently noisy, such as older adults and neurological patient populations where many factors contribute to variability in behaviour such as co-morbid age-related disorders, mood symptoms and degree of cognitive reserve^79,80^. In future work this variability could be addressed in two ways: either by incorporating peripheral physiological measures such as eye-tracking to improve the sensitivity and the specificity of the outcome measures, or by extending testing to much larger samples (such as through multi-site collaborations or web-based testing) in order to allow for controlling of the factors that contribute to the sample heterogeneity.

In summary, we found that patients were generally distracted by the presence of the distractors but that the degree of distraction caused by the high-value and low-value distractors was similar. Furthermore, we found no evidence to support the possibility that dopamine replacement modulates the effect of reward on automatic attention allocation. This is an important first step towards assessing the possible impairment of automatic attention allocation in patients, although several questions remain unanswered. Future work is required to determine if an insensitivity to reward during attentional processing is due to a failure of the mechanism responsible for guiding the selectivity of attention allocation, or if instead this is due to an inability to learn the stimulus-reward associations in the first place—associations that are necessary for the subsequent reward-guided allocation. An additional but unexplored possibility is that deficits could also arise from an inability to retrieve those associations at the time of attentional resource allocation. Though the end result for Parkinson’s patients is the same—an inability to automatically prioritize where attention is allocated on the basis of reward value—identifying the specific mechanism would provide insight about a possible common link between different cognitive deficits in Parkinson’s patients. For instance, a failure at the level of automatic attention allocation could explain a number of other reward-related deficits identified in Parkinson’s patients such as decision-making and memory. Future work will also be required to determine whether the insensitivity to reward that was similarly observed in older adults can be explained by the same mechanisms. The identification of a common source contributing to the different cognitive deficits of Parkinson’s disease, and possibly also to those of healthy aging, could eventually inform the development of cognitive rehabilitation approaches that could have broad impact.

## Supplementary Information

Supplementary Information.

## Data Availability

The datasets generated and analysed during the current study are available from the corresponding author on reasonable request.
