# Supplementary material for: Exploring reward-related attention selectivity deficits in Parkinson’s disease
Source: Sci Rep. 2021 Sep 21;11:18751. doi: 10.1038/s41598-021-97526-7 (PMC8455525; doi:10.1038/s41598-021-97526-7)
Supplement: Supplementary file 1 — Supplementary Information. [file 41598_2021_97526_MOESM1_ESM.pdf]

Supplementary Material

Exploring attention selectivity deficits in Parkinson's disease

Matthew J. D. Pilgrim[a], Zhen-Yi Andy Ou[a], Madeleine Sharp[a*]

[a]Department of Neurology and Neurosurgery, Montreal Neurological Institute, McGill University

Supplementary Table 1. Demographic and clinical characteristics of Parkinson's patients ON and OFF medication. This information is presented in addition to Table 1 in the main text because most, but not all patients, completed both the ON and OFF sessions. The groups presented below reflect the groups included in the analyses comparing patients ON vs. OFF. Twenty-eight of these patients were tested in both conditions.

Variable	Patients ON (N = 40)	Patients OFF (N = 35)	P-Value
**Age**	63.5 (6.4)	64.2 (5.9)	.636
**Education, years**	15.6 (3.2)	15.4 (3.3)	.813
**Disease duration, years**	4.8 (3.4)	5.1 (3.4)	.738
**Total PD Medication, mg**	674.2 (336.7)	667.0 (332.0)	.926
**% Taking Dopamine Agonists**	14%	17%	.999
**% Female**	23%	33%	.503
**MoCA**	27.1 (2.2)	27.0 (2.5)	.927
**Verbal Fluency (MoCA)**	12.4 (4.3)	12.6 (4.0)	.873
**Digit Span Test**	11.2 (2.3)	11.2 (2.1)	.961
**Symbol Digit Modalities Test**	40.9 (10.5)	40.7 (10.5)	.923
**Geriatric Depression Scale**	7.9 (6.1)	7.6 (5.8)	.815
**Apathy Evaluation Scale**	58.5 (8.01)	58.6 (7.9)	.945

MoCA = Montreal Cognitive Assessment, Verbal Fluency is taken from the Language section of the MoCA. Values presented are mean (SD).

Supplementary Table 2. Reward Association phase accuracy model coefficient estimates
Fixed effects estimates obtained from the mixed effects regression indicating the influence of trial type, medication or disease, and other covariates on response accuracy during the Reward association phase**.** Coefficients are presented separately for the three models that were run: PD Only to measure the effect of medications, HC Only, and All Subs to measure the difference between patients and controls. Models with and without covariates (session, education, SDMT, as applicable) are shown.

Fixed effect	Model	Estimate (SE)	P-Value
Models with covariates			
**Intercept**	PD Only	0.038 (0.370)	0.918
**Medication**	PD Only	0.005 (0.063)	0.938
**Reward Level**	PD Only	0.019 (0.031)	0.553
**Medications * Reward Level**	PD Only	0.021 (0.024)	0.368
**Session**	PD Only	0.042 (0.064)	0.513
**SDMT**	PD Only	0.042 (0.009)	<0.001 ***
**Intercept**	HC Only	1.518 (0.824)	0.056
**Reward Level**	HC Only	0.006 (0.053)	0.907
**Session**	HC Only	0.091 (0.090)	0.312
**SDMT**	HC Only	0.022 (0.017)	0.190
**Intercept**	All Subs	0.073 (0.501)	0.885
**Disease**	All Subs	-0.289 (0.087)	<0.001 ***
**Reward Level**	All Subs	-0.004 (0.020)	0.8256
**Disease * Reward Level**	All Subs	0.029 (0.020)	0.136
**Session**	All Subs	0.054 (0.0529)	0.308
**SDMT**	All Subs	0.032 (0.008)	<0.001 ***
**Education**	All Subs	0.041 (0.027)	0.121
Models without covariates			
**Intercept**	PD Only	1.950 (0.140)	<0.001 ***
**Medication**	PD Only	0.022 (0.079)	0.780
**Reward Level**	PD Only	-0.013 (0.044)	0.775
**Medications*Reward Level**	PD Only	-0.004 (0.030)	0.903
**Intercept**	HC Only	2.679 (0.157)	<0.001 ***
**Reward Level**	HC Only	0.035 (0.056)	0.533
**Intercept**	All Subs	2.290 (0.105)	<0.001 ***
**Disease**	All Subs	-0.381 (0.105)	<0.001 ***
**Reward Level**	All Subs	0.010 (0.035)	0.777
**Disease*Reward Level**	All Subs	-0.019 (0.035)	0.582

SDMT = Symbol Digit Modalities Test

Supplementary Table 3. Reward Association phase reaction time model coefficient estimates
Fixed effects estimates obtained from the mixed effects regression indicating the influence of trial type, medication or disease, and other covariates on reaction time (log(RT)) during the Reward association phase**.** Coefficients are presented separately for the three models that were run: PD Only to measure the effect of medications, HC Only, and All Subs to measure the difference between patients and controls.

Fixed effect	Model	Estimate (SE)	P-Value
**Intercept**	PD Only	-0.020 (0.020)	0.312
**Medication**	PD Only	-0.002 (0.003)	0.591
**Reward Level**	PD Only	0.001 (0.003)	0.623
**Medications * Reward Level**	PD Only	0.002 (0.002)	0.420
**Session**	PD Only	0.002 (0.003)	0.435
**SDMT**	PD Only	-0.002 (0.000)	0.001 **
**Intercept**	HC Only	0.009 (0.041)	0.833
**Reward Level**	HC Only	-0.004 (0.002)	0.091
**Session**	HC Only	-0.001 (0.002)	0.619
**SDMT**	HC Only	-0.002 (0.001)	0.008**
**Intercept**	All Subs	0.012 (0.030)	0.683
**Disease**	All Subs	0.005 (0.005)	0.283
**Reward Level**	All Subs	-0.001 (0.002)	0.415
**Disease * Reward Level**	All Subs	0.002 (0.002)	0.133
**Session**	All Subs	0.0004 (0.002)	0.839
**SDMT**	All Subs	-0.002 (0.000)	<0.001 ***
**Education**	All Subs	-0.0003 (0.001)	0.828
**Sex**	All Subs	-0.015 (0.009)	0.105

SDMT = Symbol Digit Modalities Test

Supplementary Table 4. Mean reaction times during the Attention test phase.
Mean reactions times (in seconds) are presented for the three trial types (no distractor, low reward and high reward) for the three groups. In the case of the Control group reaction times are averaged across both sessions. Vales are mean (SD).

Group	Medication Status	Reward Level	Reaction Time (s)
**Control**	NA	None	0.871 (0.230)
**Control**	NA	Low	0.875 (0.233)
**Control**	NA	High	0.881 (0.235)
**Parkinson's**	OFF	None	0.894 (0.237)
**Parkinson's**	OFF	Low	0.907 (0.237)
**Parkinson's**	OFF	High	0.895 (0.235)
**Parkinson's**	ON	None	0.905 (0.236)
**Parkinson's**	ON	Low	0.916 (0.237)
**Parkinson's**	ON	High	0.908 (0.236)

Supplementary Table 5. Attention Test phase – reaction time model coefficient estimates.
Fixed effects estimates obtained from the mixed effects regression indicating the influence of trial type, medication or disease, and other covariates on the reaction times (log(RT)) during the Attention Test phase. Coefficients are presented separately for the three models that were run: PD Only to measure the effect of medications, HC Only, and All Subs to measure the difference between patients and controls. Models with and without covariates (session, education, sex, SDMT, as applicable) are shown.

Fixed effect	Model	Estimate (SE)	P-Value
Models with covariates			
**Intercept**	PD Only	0.033 (0.026)	0.224
**Reward Vector 1**	PD Only	0.004 (0.001)	0.002 **
**Reward Vector 2**	PD Only	0.001 (0.014)	0.553
**Medication**	PD Only	0.008 (0.003)	0.006 **
**Reward Vector 1* Medication**	PD Only	-0.000 (0.001)	0.840
**Reward Vector 2* Medication**	PD Only	0.001 (0.001)	0.373
**Session**	PD Only	-0.006 (0.003)	0.021 *
**SDMT**	PD Only	-0.002 (0.001)	<0.001 ***
**Intercept**	HC Only	0.014 (0.047)	0.772
**Reward Vector 1**	HC Only	-0.000 (0.001)	0.838
**Reward Vector 2**	HC Only	0.002 (0.001)	0.163
**Session**	HC Only	-0.016 (0.002)	<0.001 ***
**SDMT**	HC Only	-0.002 (0.001)	0.089
**Intercept**	All Subs	0.010 (0.033)	0.751
**Reward Vector 1**	All Subs	0.002 (0.001)	0.041 *
**Reward Vector 2**	All Subs	0.001 (0.001)	0.163
**Disease**	All Subs	0.001 (0.006)	0.863
**Reward Vector 1 * Disease**	All Subs	0.002 (0.001)	0.019 *
**Reward Vector 2 * Disease**	All Subs	-0.001 (0.001)	0.585
**Session**	All Subs	-0.012 (0.002)	<0.001 ***
**SDMT**	All Subs	-0.002 (0.001)	<0.001 ***
**Education**	All Subs	0.002 (0.002)	0.193
**Sex**	All Subs	-0.017 (0.011)	0.137
Models without covariates			
**Intercept**	PD Only	-0.057 (0.007)	<0.001 ***
**Reward Vector 1**	PD Only	0.004 (0.001)	0.002 **
**Reward Vector 2**	PD Only	0.001 (0.001)	0.551
**Medication**	PD Only	0.008 (0.003)	0.005 **
**Reward Vector 1*Medication**	PD Only	-0.000 (0.001)	0.848
**Reward Vector 2*Medication**	PD Only	0.001 (0.001)	0.380
**Intercept**	HC Only	-0.067 (0.009)	<0.001 ***
**Reward Vector 1**	HC Only	-0.001 (0.001)	0.479
**Reward Vector 2**	HC Only	0.002 (0.001)	0.115
**Intercept**	All Subs	-0.062 (0.006)	<0.001 ***
**Reward Vector 1**	All Subs	0.002 (0.001)	0.110
**Reward Vector 2**	All Subs	0.002 (0.001)	0.137
**Disease**	All Subs	0.005 (0.006)	0.360
**Reward Vector 1*Disease**	All Subs	0.003 (0.001)	0.013 *
**Reward Vector 2*Disease**	All Subs	-0.001 (0.001)	0.479

Supplementary Table 6. Attention test phase reaction time analysis to look at effect of reward level and disease/medication. Difference estimates computed from weighted sums of the regression coefficients presented in Supplementary Table 4 in order to obtain the effect of trial type on reaction times, and the difference between groups for this effect.

Trial type compared	Source Model	Difference estimate (SE)	P-Value
**Low vs High * medication**	PD Only	-0.002 (0.003)	0.546
**Low vs None * medication**	PD Only	0.001 (0.002)	0.757
**High vs None * medication**	PD Only	0.002 (0.002)	0.313
**Low vs High**	HC Only	-0.002 (0.003)	0.376
**Low vs None**	HC Only	0.001 (0.002)	0.530
**High vs None**	HC Only	0.004 (0.002)	0.100
**Low vs High * Disease**	All Subs	0.003 (0.002)	0.115
**Low vs None * Disease**	All Subs	0.004 (0.002)	0.008 **
**High vs None * Disease**	All Subs	0.001 (0.002)	0.423

Supplementary Table 7. Attention Test phase accuracy model coefficient estimates.
Fixed effects estimates obtained from the mixed effects regression indicating the influence of trial type, medication or disease, and other covariates on accuracy during the Attention Test phase. Coefficients are presented separately for the three models that were run: PD Only to measure the effect of medications, HC Only, and All Subs to measure the difference between patients and controls.

Fixed effect	Model	Estimate (SE)	P-Value
**Intercept**	PD Only	-0.007 (0.440)	0.987
**Reward Vector 1**	PD Only	-0.005 (0.038)	0.890
**Reward Vector 2**	PD Only	-0.053 (0.038)	0.160
**Medication**	PD Only	-0.048 (0.047)	0.306
**Reward Vector 1* Medication**	PD Only	0.055 (0.038)	0.145
**Reward Vector 2* Medication**	PD Only	-0.048 (0.038)	0.202
**Session**	PD Only	0.161 (0.050)	0.001 **
**SDMT**	PD Only	0.050 (0.011)	<0.001 ***
**Intercept**	HC Only	1.322 (0.695)	0.057
**Reward Vector 1**	HC Only	-0.035 (0.050)	0.475
**Reward Vector 2**	HC Only	0.003 (0.050)	0.946
**Session**	HC Only	0.173 (0.034)	<0.001 ***
**SDMT**	HC Only	0.023 (0.014)	0.068
**Intercept**	All Subs	0.147 (0.516)	0.776
**Reward Vector 1**	All Subs	-0.016 (0.034)	0.634
**Reward Vector 2**	All Subs	-0.024 (0.034)	0.474
**Disease**	All Subs	-0.073 (0.096)	0.445
**Reward Vector 1 * Disease**	All Subs	0.015 (0.033)	0.652
**Reward Vector 2 * Disease**	All Subs	-0.031 (0.034)	0.350
**Session**	All Subs	0.179 (0.034)	<0.001 ***
**SDMT**	All Subs	0.039 (0.009)	<0.001 ***
**Education**	All Subs	0.034 (0.027)	0.208
**Sex**	All Subs	-0.231 (0.177)	0.193

SDMT = Symbol Digit Modalities Test

Supplementary Table 8. Attention test phase analysis to look at effect of overall distraction – model estimates.

Fixed effects estimates obtained from the mixed effects regression indicating the influence of trial type, medication or disease, and other covariates on the reaction times (log(RT)) during the Attention Test phase. Coefficients are presented separately for the three models that were run: PD Only to measure the effect of medications, HC Only, and All Subs to measure the difference between patients and controls.

Fixed effect	Model	Estimate (SE)	P-Value
**Intercept**	PD Only	0.031 (0.026)	0.241
**Medication**	PD Only	0.007 (0.003)	0.007 **
**Distractor Presence**	PD Only	0.004 (0.001)	<0.001 ***
**Medication * Distractor Presence**	PD Only	0.001 (0.001)	0.421
**Session**	PD Only	-0.006 (0.003)	0.002 *
**SDMT**	PD Only	-0.002 (0.001)	<0.001 ***
**Intercept**	All Subs	0.010 (0.033)	0.767
**Disease**	All Subs	0.001 (0.006)	0.923
**Distractor Presence**	All Subs	0.003 (0.001)	<0.001 ***
**Disease * Distractor Presence**	All Subs	0.001 (0.001)	0.035
**Session**	All Subs	-0.012 (0.002)	<0.001 ***
**SDMT**	All Subs	-0.002 (0.001)	<0.001 ***
**Education**	All Subs	0.002 (0.002)	0.195
**Sex**	All Subs	-0.017 (0.011)	0.136

SDMT = Symbol Digit Modalities Test

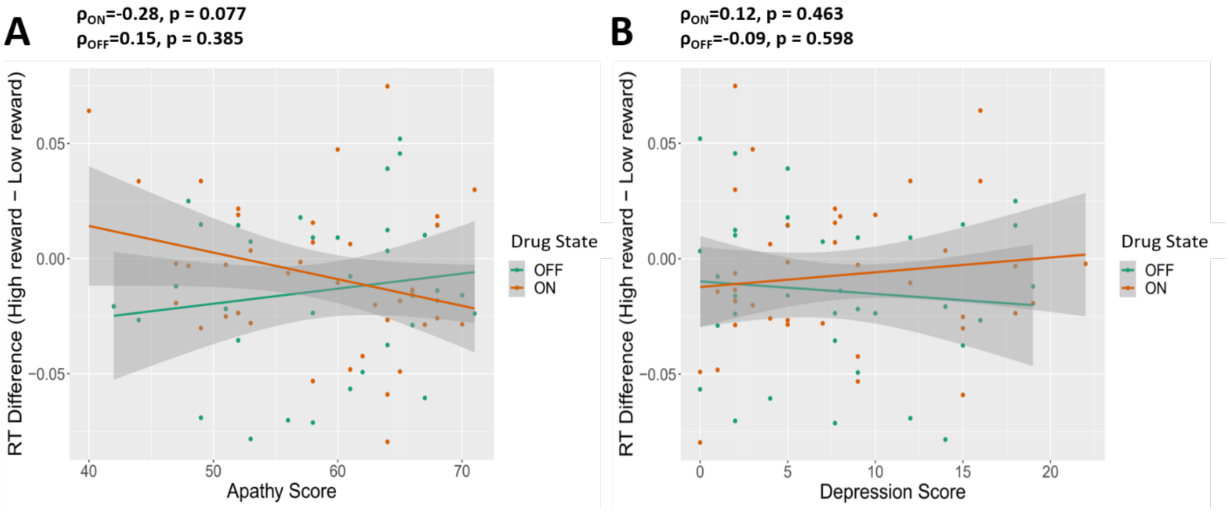

Supplementary Figure 1. Individual differences in reward driven attention allocation are not related to apathy or depression in Parkinson's patients. The effect of reward on attention, measured as the difference in reaction time in seconds between trials with a high reward distractor versus trials with a low reward distractor, is plotted separately for performance when tested OFF and ON dopaminergic medications, and is plotted against scores on the Apathy Evaluation Scale scores (A) and Geriatric Depression Scale (B).
